# Performance Assessment of the Geometrical Parameters of a Cavity Receiver for a Thermochemical Reactor in a Solar Tower Plant

**DOI:** 10.1021/acsomega.5c13658

**Published:** 2026-06-17

**Authors:** Diana E. Rodríguez-Sánchez, Camilo A. Arancibia-Bulnes, Luis P. Ramirez-Rodríguez, Rodolfo Peón-Anaya, Julio Waissman-Vilanova, David Riveros-Rosas

**Affiliations:** † Instituto de Energías Renovables, 418104 Universidad Nacional Autónoma de México, Privada Xochicalco s/n, Col. Centro, Temixco 62580, Morelos, Mexico; ‡ Departamento de Física, 27813 Universidad de Sonora, Blvd. Luis Encinas Johnson s/n, 83000 Hermosillo, Sonora, Mexico; § Departamento de Ingeniería Química, Universidad de Sonora, Blvd. Luis Encinas Johnson s/n, 83000 Hermosillo, Sonora, Mexico; ∥ Departamento de Matemáticas, Universidad de Sonora, Blvd. Luis Encinas Johnson s/n, 83000 Hermosillo, Sonora, Mexico; ⊥ Instituto de Geofísica, 7180 Universidad Nacional Autónoma de México, Av. Universidad 3000, 04510 Ciudad de México, Mexico

## Abstract

Water splitting in thermochemical reactors, driven by concentrated solar energy, represents a promising and truly sustainable method for producing renewable hydrogen. However, the current efficiency of solar-to-hydrogen energy conversion indicates that significant technological improvements are needed before this process can become commercially viable on an industrial scale. To contribute to the advancement of thermochemical reactor design, this work evaluated different tube configurations inside a cavity receiver for a two-stage redox cycle driven by a 1.5 MWth solar tower plant. Specifically, six different configurations of 80 tubes were considered within a single-cavity receiver. The thermal requirements for the reduction step were met by changing the geometry of the passive reactors. Simulations indicated that multiple tube arrays can exceed the critical temperature of 1300 °C necessary for thermal reduction. Additionally, a closely spaced, single-layer configuration was found to be the most effective in minimizing temperature gradients within a chamber, which is essential to achieve uniform reaction conditions. This study demonstrates that an effective design of the internal tube layout is essential to control the complex thermal environment in solar cavity reactors and presents feasible configurations for solar hydrogen production in solar tower plants.

## Graphical abstract

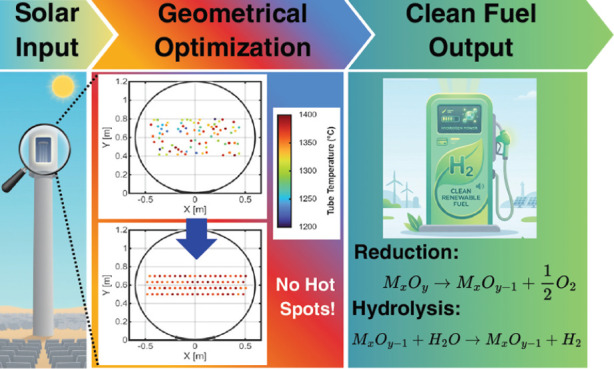

## Introduction

Hydrogen is a versatile energy carrier and a crucial feedstock for major industrial sectors, including petroleum refining, fertilizer production, plastics, pharmaceuticals, and food.^1^ As a clean fuel, hydrogen’s only combustion byproduct is water, making it a key component of future energy systems. Although hydrogen is the most abundant element in the universe, it primarily exists on Earth in compounds such as water and hydrocarbons. Consequently, isolating hydrogen requires energy-intensive chemical processes. Currently, more than 95% of global hydrogen production is based on fossil fuels, a process that emits approximately 830 million tons of carbon dioxide each year.^2^ This reliance on traditional methods presents a significant barrier to achieving climate change mitigation goals. Fortunately, solar energy, the most abundant renewable resource on Earth, offers promising pathways for sustainable hydrogen production. Solar-driven technologies, particularly solar thermal routes, are essential for the development of future clean energy solutions and can generate molecular hydrogen with a minimal carbon footprint.^3,4^

The conversion of solar energy into hydrogen can be achieved through three primary methods: electrochemical, photochemical, and thermochemical routes.^5,6^ The electrochemical approach uses electricity generated from solar sources, such as photovoltaic modules or thermal power plants, to drive water electrolysis, directly converting water into hydrogen and oxygen.^7,8^ Alternatively, in the photochemical method, semiconductor photocatalysts are employed. When illuminated, they can directly split water or steam/*CO*_2_ mixtures to produce hydrogen (*H*_2_) or syngas, effectively mimicking natural photosynthesis at a molecular level.^3,9^ Finally, solar thermochemical methods use high-temperature process heat from concentrated solar thermal (CST) systems to drive endothermic reactions. In particular, two-step metal oxide redox cycles show promise for efficient and large-scale hydrogen production.^5,6^ Each of these methods presents unique advantages and drawbacks in converting solar energy into hydrogen fuel.

Concentrated solar thermal systems generate hydrogen through five primary pathways: thermolysis, thermochemical cycles, cracking, reforming, and gasification.^5^ These pathways can be categorized into two groups. The first group includes methods that extract hydrogen from carbon-based feedstocks, such as cracking, reforming, and gasification, which can produce greenhouse gas emissions.^6^ The second group consists of direct water-splitting methods, namely thermolysis and thermochemical cycles, which provide a carbon-free route to hydrogen production. However, direct thermolysis, the simplest water-splitting method, is impractical due to its requirement for extreme temperatures (above 2200 °C), which place significant stress on reactor materials.^10,11^

Thermochemical water splitting (TCWS) addresses this challenge by employing a reusable metal oxide in a two-step redox cycle at much lower temperatures (800–1500 °C).^12^ The process operates as follows:

1) Reduction Step (High Temperature): The metal oxide is heated with concentrated solar energy, causing it to release pure oxygen (O_2_) while undergoing a chemical reduction.

2) Oxidation Step (Lower Temperature): The reduced metal oxide is then exposed to steam (H_2_O). It avidly extracts oxygen from water, reverting to its original state (reoxidized) and releasing pure hydrogen (H_2_) in the process.

This cyclical redox process offers several key advantages: hydrogen and oxygen are produced in separate stages, preventing explosive mixtures and product recombination;^13^ the metal oxide is continuously regenerated, resulting in oxygen being the only byproduct of this clean, closed-loop system^14^ and it has a high theoretical solar-to-fuel efficiency, potentially around 52%.^6^ Given these features, solar thermochemical water-splitting cycles are particularly attractive, providing a direct pathway to convert heat into fuel and becoming a primary focus of research on green hydrogen.^15^

An efficient solar reactor is essential for conducting thermochemical cycles. Solar reactors are typically designed as cavity receivers that capture concentrated solar radiation through an aperture.^16^ The design must minimize reradiation and reflection losses through this aperture.^17^ Reactors are classified as either directly or indirectly irradiated. In direct reactors, reactants are exposed to solar flux, often requiring a transparent quartz window that can be a technical challenge under high pressures and temperatures,^5,6,18^ In indirectly irradiated reactors, which are the focus of this work, radiation is absorbed by surfaces, such as ceramic tubes, which then transfer heat to the reactants.^16,19^

In recent decades, significant efforts have focused on demonstrating the technical feasibility of scaling these technologies from laboratory tests to pilot-scale experimental facilities. Table 1 presents key real-world implementations of solar receivers, detailing their scales, receiver types, and operational temperatures. For example, testing of directly irradiated receivers, such as the 50 kWth cavity-receiver with ceria reticulated porous ceramic (RPC) foam at IMDEA Energy, demonstrated stable cycling and peak temperatures of 1775 K for two-step redox cycles; however, maintaining temperature uniformity remains a structural challenge.^20^ The 300 kWth SOLZINC beam-down facility also achieved high temperatures but exhibited temperature gradients, with the upper part of the chamber being 50–100 K cooler.^21^ For indirectly irradiated systems, pilot-scale facilities such as the 100 kWth SSPS-CRS and the SolH2 multitubular cavity reactors reached operational temperatures near 1473 K, with average temperature deviations of up to 140 K across their tube arrays.^22,23^ The 25 kWth HoSIER cubic multitubular reactor achieved 1430 K but showed a Gaussian flux distribution concentrated at the center.^24^ These experimental studies indicate that engineering a single-cavity receiver to achieve the high temperatures required for the initial reduction step, while minimizing nonuniform temperature distributions, remains a persistent challenge. To address these thermal gradients before physical construction, modeling of indirectly irradiated reactors is essential for optimizing performance.

**Table 1:** Overview of Solar Receiver and Reactor Systems

System/Solar Scale	Receiver Type	Max Operating Temp. Achieved	Temperature Distribution Quality	Redox Material/Cycle
SolH2 100 kW^22^	Cavity with packed-bed multitubular array (80 ferrite-coated alumina tubes)	1473 K	Relatively homogeneous, with tube temperature deviations ≤ 100–120 K	NiFe_2_O_4_/ZrO_2_ ferrite cycle
ETH-PSI 50 kW^25^	Cavity-receiver with directly irradiated ceria RPC (CeO_2_)	1762 K	ΔT improved from 377 to 113 K (switching from a 10 ppi to a 3 ppi larger-pore structure)	CeO_2_ redox with temp/pressure swing
SSPS-CRS 100 kW (80 kW delivered)^23^	Multitubular cavity reactor	1451 K	Average tube temperature deviations of 120–140 K	Mixed ferrites thermochemical cycle for H_2_
HoSIER (UNAM) 25 kW^24^	Cubic cavity multitubular solar reactor	1430 K	Heat is highly concentrated at the middle of tubes	Hydrocarbon gasification for H_2_ and synthesis gas
MWSF (CNRS) 90 to 128 kW^26^	Cavity-based with a rotating reactor design	>2000 K	Uniform high-temperature environment with high thermal efficiency	Zn/ZnO thermochemical cycle
SOLZINC 300 kW^21^	Beam-down, two-cavity (upper absorber shield; lower packed-bed reaction chamber)	1300–1500 K	Chamber temperature deviations of 50–100 K; high temperature gradients through the packed bed	Carbothermic reduction of ZnO
Hydrosol 45 kW^27^	Monolithic honeycomb structures coated with reactive metal oxides	1473 K	Inhomogeneous temperature distribution across the absorber	Two-step mixed iron oxide water-splitting
KIER 40 kW^28^	Volumetric ceramic foam device (CeO_2_ coated)	1873 K	Temperature gradient on the foam device Surface was 150 K	Two-step thermochemical water-splitting using CeO_2_
IMDEA Energy 50 kW^20^	Cavity-receiver with a directly exposed ceria RPC structure	1775 K	Stable cycling achieved; maintaining temperature uniformity was challenging	Two-step thermochemical redox cycle using CeO_2_
Niigata Univ 30 kW^29^	Beam-down, directly irradiated fluidized bed reactor	∼1500 K	Fountain periphery is hotter than the core due to high-velocity convection	Two-step water-splitting using nonvolatile metal oxides

Previous studies have used 2D steady-state models to compute temperature and reaction rates,^19^ analyzed the impact of windowed vs windowless designs,^30^ and developed heliostat aiming strategies to homogenize flux.^31^ However, a key challenge remains the highly nonuniform temperature distribution that arises in multitube arrays.^32,33^ While some models have optimized small arrays of tubes,^34^ 3D steady-state models for large, optimized tube arrays are still scarce.^35,36^

The advancement of solar reactor design is essential for enhancing the efficiency of solar-to-hydrogen energy conversion.^4,37^ This study seeks to contribute to ongoing efforts aimed at improving reactor performance by optimizing cavity receivers for multicycle operation^22^ and enhancing the efficiency of solar radiation capture through better mirror reflectors, reduced tracking errors, and precision optics. Additionally, this work seeks to support current research efforts, which include hybrid cycles as well as the testing of new oxides and processes, by tailoring the thermal requirements for each application.

To achieve this, we present a steady-state computational heat transfer model to optimize tube distributions within a multitubular solar reactor, based on the geometry described by Gonzalez-Pardo et al.^23,31^ and Vidal.^22^ The model accounts for radiative and convective heat losses using a Monte Carlo method and Nusselt number correlations, respectively.

In this context, the main objective of this work is to identify combinations of tube arrangements inside the cavity receiver that can achieve the distinct temperatures required for the two-step thermochemical cycle (e.g., > 1300 °C for the reduction stage). Conventional operations achieve the required 1300 °C and flux uniformity by dynamically modulating the heliostat field, intermittently aiming specific groups of mirrors to manage thermal loads.^23,31^ This study examines a passive structural approach. We investigate whether optimizing the geometric configuration of the absorber tubes within a single-cavity receiver can achieve comparable thermal performance and uniformity while maintaining total field utilization. This approach assesses the feasibility of creating the specific thermal environment required for the high-temperature reduction step.

## Methodology

### System Description

The system examined in this study is a conceptual 1.5 MWth central tower plant, modeled under the solar resource conditions of Hermosillo, Mexico (29.07^◦^ N, 110.96^◦^ W). This configuration serves as a numerical case study for performance assessment; the geographical parameters and Direct Normal Irradiation (DNI) profiles are representative of this region, which is a prime candidate for high-temperature solar applications. The receiver is designed to absorb concentrated solar radiation from a heliostat field and transfer it to an array of tubular reactors where the thermochemical reactions occur.

### Receiver Geometry and Materials

The receiver is a cylindrical thermally insulated cavity that is 1.2 m high and has a radius of 0.6 m as shown in Figure 1. On the front face, there is a semiarched rectangular aperture measuring 0.46 m by 0.46 m, which allows concentrated solar flux to enter the receiver. All interior surfaces of the cavity are made of Silicon Carbide (SiC). For this study, we assumed an emissivity (ϵ_*sic*_) and absorptivity (α_*sic*_) of 0.9 for SiC, consistent with the values used by Tapia et al.^23,38–40^

**Figure 1:**
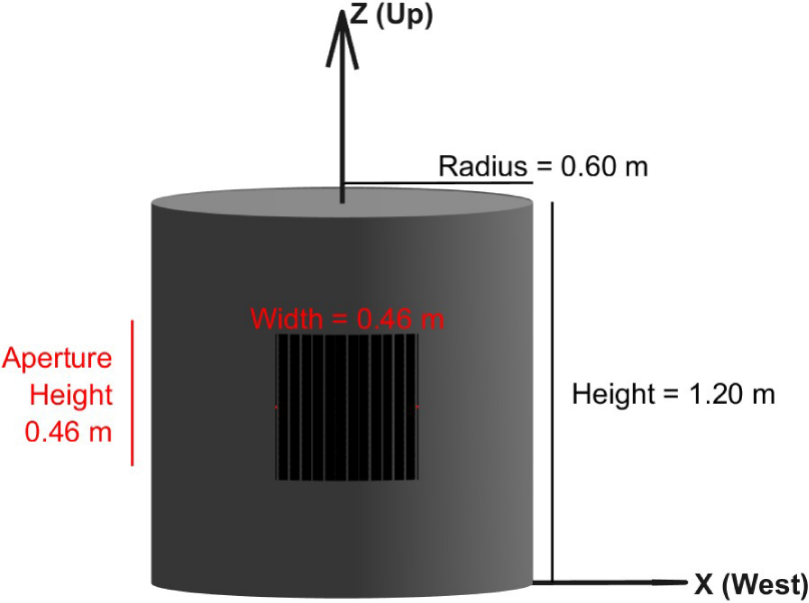
Receiver dimensions.

### Reactor Tube Configurations

Inside the receiver, 80 vertical reactor tubes are placed to absorb thermal energy. These tubes are constructed from Alumina, each measuring 1.2 m in length and having an external radius of 1.26 cm. For Alumina, we assumed an emissivity (ϵ_*al*_) and absorptivity (α_*al*_) of 0.3, a representative value found in the literature, including the works of Henninger and Tapia.^23,41^ To explore the impact of passive geometric design on thermal performance, this study analyzes six different tube configurations (TC), as illustrated in Figure 2. Each configuration represents a unique spatial arrangement of the 80 tubes within the cavity, with the main goal of identifying a layout that optimizes the temperature distribution for the intended thermochemical process.

**Figure 2:**
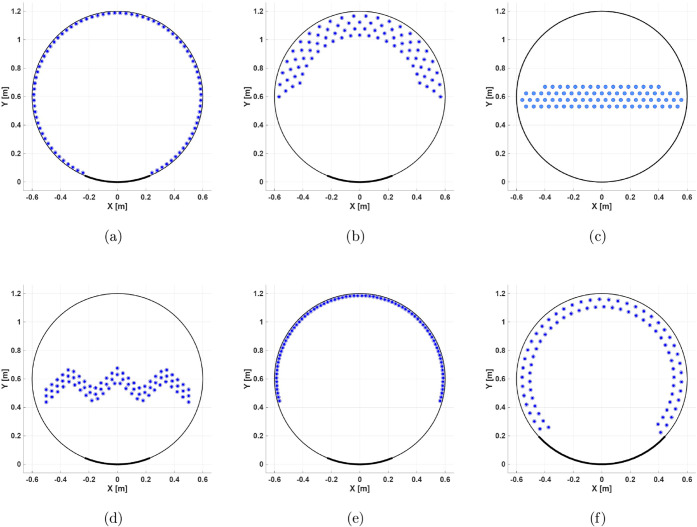
Tube configuration inside the cavity. (a) TC1, (b) TC2, (c) TC3, (d) TC4, (e) TC5, and (f) TC6.

### Heliostat Field Design

The solar heliostat field was designed using the Windelsol software package, a graphical user interface for the DeLsol3 code developed by Sandia National Laboratories.^42^ The design was configured for the plant’s location in Hermosillo, Mexico, targeting a vertical flat-plate receiver on a 30 m tower. The resulting north-facing field consists of 1,100 spherically canted heliostats, each measuring 1.2 m × 1.2 m. These are distributed over a ground area 83 m deep and 65 m wide. The central aiming point for all heliostats is the geometric center of the receiver’s aperture (0, 0, 30).

### Operational Parameters and Power

The analysis was performed using a Direct Normal Irradiance (DNI) of 1000 W/m^2^, a heliostat reflectivity of 0.91, and an average optical error of 2 mrad. For this study, we selected all 1,100 heliostats (see Figure 3), which provides a peak thermal input of approximately 1.25 MWth to the receiver aperture.

**Figure 3:**
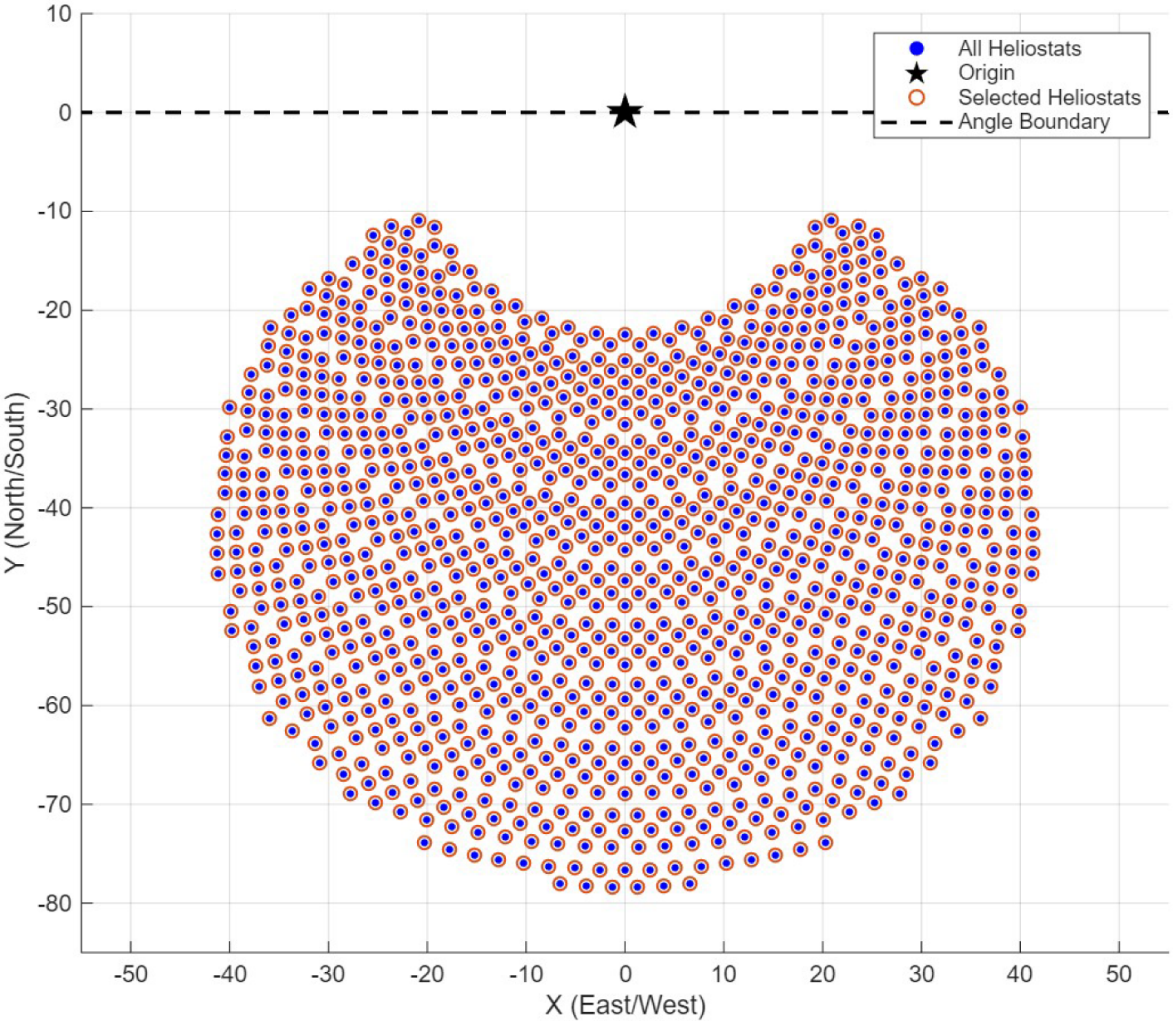
Heliostat field section.

Although the receiver geometry is based on the designs of Gonzalez-Pardo et al. and Tapia^23,31^ to ensure a consistent basis for comparison, our study employs a different solar field configuration and a lower thermal power input (1.5 MWth compared to 2 MWth). This distinction is important, as our analysis specifically targets the thermal performance resulting from this power level and aiming strategy.

This configuration ensures that the required thermal power input is delivered to the receiver aperture and serves as a foundational step in a larger optical optimization effort aimed at reducing the capital costs of industrial-scale hydrogen production. Future work will explore ways to minimize the required number of heliostats by incorporating emerging technologies, such as low-cost relocatable heliostats^43^ and pattern-free layouts that eliminate seasonal shading.^44^

### Heliostat Field Ray Tracing

The spatial and angular distribution of solar radiation at the receiver’s aperture plane was determined using a Monte Carlo ray tracing (MCRT) model developed in MATLAB. In this model, 1 × 10^5^ rays were traced from each heliostat to the receiver. For a full-field simulation using all 1,100 heliostats, this amounts to a total of 1.1 × 10^8^ rays, ensuring a high-resolution map of the resulting solar flux. The radiative power assigned to each ray is proportional to the heliostat area and the incident solar irradiance.

### Optical Modeling and Mathematical Formulation

The reflection of solar rays from the heliostat surfaces is modeled as quasi-specular. A circular Gaussian angular dispersion is applied around the ideal specular direction to account for optical imperfections. This ″degraded Sun″ model^45^ combines the angular distribution of the Sun (σ_sun_) with the heliostat’s slope (σ_slope_) and specularity (σ_spec_) errors into a single total standard deviation, σ_tot_:

1 $$\sigma_{tot}=\sqrt{4\sigma_{slope}^{2}+\sigma_{spec}^{2}+\sigma_{sun}^{2}}$$

The ideal specular reflection vector **r̂** is calculated based on the incoming sun vector **ŝ** and the mirror normal vector **n̂**:

2 r̂=−ŝ+2(ŝ·n̂)n̂

The final deviated reflection vector **r̂**’ is then generated by applying a random angular perturbation to the ideal vector **r̂**:

3 $${\mathrm{r̂}}^{\prime }=\cos\theta_{r}\mathrm{r̂}+\sin\theta_{r}\cos\phi_{r}{\mathrm{t̂}}_{1}+\sin\theta_{r}\sin\phi_{r}{\mathrm{t̂}}_{2}$$

The random azimuthal angle ϕ_*r*_ and polar angle θ_*r*_ are sampled from their respective distributions using uniformly distributed random numbers, *R*_ϕ_ and *R*_θ_ (where 0 ≤ R ≤ 1):

4 $$\phi_{r}=2\pi R_{\phi }$$

and

5 $$\theta_{r}={\left[-2\sigma_{tot}^{2}\ln(1-R_{\theta })\right]}^{1/2}$$

The unit vectors **t̂**_1_ and **t̂**_2_, which are perpendicular to the specular vector **r̂**, are calculated as

6 $${\mathrm{t̂}}_{1}=\frac{\mathrm{n̂}\times \mathrm{r̂}}{\left|\mathrm{n̂}\times \mathrm{r̂}\right|}$$

7 $${\mathrm{t̂}}_{2}=\frac{{\mathrm{t̂}}_{1}\times \mathrm{r̂}}{\left|{\mathrm{t̂}}_{1}\times \mathrm{r̂}\right|}$$

All ray-tracing simulations were conducted for the specific conditions of the summer solstice (June 21st) at solar noon. A Direct Normal Irradiance (DNI) of 1000 *W*/*m* ^2^ was assumed for all cases.

### Heat Transfer Model

The thermal performance of the solar reactor is evaluated using a steady-state heat transfer model, which is designed to determine the temperature of each component within the receiver.

#### Model Formulation and Assumptions

A steady-state approach was selected over a transient analysis because the latter is computationally intensive, requiring significant processing time and data storage resources. Additionally, the high thermal inertia of cavity receivers reduces the effects of short-term solar fluctuations, making a steady-state analysis a valid representation of the system’s behavior under critical operating conditions,^19,23,31,46,55^

To facilitate a high-fidelity thermal analysis of the system, the interior of the receiver was extensively discretized into a refined mesh of 6,242 distinct isothermal surface elements. Unlike lumped-parameter models, this simulation calculates the incident solar flux, view factors, radiative emissions, and resulting temperature distribution for each individual mesh element. The discretization strategy is defined as follows:

- Cylindrical Cavity Walls: The inner wall is divided into three primary regions: the top cap, the bottom cap, and the lateral wall. The lateral wall is further discretized into a grid of 12 axial (height) segments by 40 angular (circumferential) sectors, resulting in 482 elements for the cavity enclosure.
- 80 Tubes: To accurately capture temperature gradients, the surface of each of the 80 inner tubes is discretized into 12 axial segments by 6 angular sectors. This yields 5,760 tube elements in total.

Consequently, the simulation solves the energy balance and surface temperature for every individual surface element defined by this mesh, allowing for precise determination of localized thermal hotspots and radiative exchange.

The model operates under the following key assumptions:

- All interior surfaces are considered opaque, gray (where emissivity equals absorptivity, (ϵ = α), and behave as diffuse emitters and reflectors.
- The gas within the cavity (air) does not engage in radiation exchange; it is treated as nonabsorbing and nonemitting.
- The receiver aperture is regarded as an open hole and is modeled as a blackbody surface at ambient temperature when calculating radiation losses.

#### Energy Balance Formulation

A net energy balance is established for each surface element, designated as ″*j*″. To achieve thermal equilibrium for a specified surface ″*j*″, the total energy absorbed must equal the total energy lost. *Irradiance* (*H*_*j*_) is defined as the total radiation heat flux incident on surface ″*j*″ from all sources, measured per unit area [W/m^2^]. Conversely, *radiosity* (*B*_*j*_) represents the total radiation heat flux emitted from surface ″*j*″ per unit area [W/m^2^]. This includes both the radiation emitted by the surface itself and any radiation that is reflected by it.^47^ Consequently, the energy balance equation for a given surface ″*j*″ can be expressed in terms of irradiance and radiosity as follows:

8 $$H_{j}\equiv B_{j}+q_{{\mathrm{net}}_{j}}$$

In this particular case, the previous equation can be rewritten as

9 $$Q_{{\mathrm{solar}}_{j}}+Q_{{\mathrm{rad}}_{j}}=Q_{em_{j}}+q_{{\mathrm{net}}_{j}}$$

Where $Q_{\mathrm{solar}j}$ is the absorbed concentrated solar radiation from the heliostat field, $Q_{\mathrm{rad}j}$ is the thermal radiation incident on surface ″*j*″ from all other interior surfaces ″*i*″, $Q_{\mathrm{em},j}$ is the thermal radiation emitted by surface ″*j*″ and $q_{\mathrm{net}j}$ is the sum of all convective heat losses from surface ″*j*″.

#### Heat Transfer Term Definitions

Each term in the energy balance is defined as follows:

1 Absorbed Solar Radiation $(Q_{\mathrm{solar}j})$ is calculated using the results from the MCRT simulation.
10 $$Q_{{\mathrm{solar}}_{j}}=\mu N_{h}P_{h_{j}}$$
  Where $P_{h_{j}}$ is the fraction of total solar power absorbed by surface ″*j*″ and μ_*Nh*_ is the radiative power of each of the *N*_*h*_ rays reflected from the heliostat field.
2 Incident Radiation $(Q_{\mathrm{rad}j})$ is the total energy emitted by all other surfaces ″*i*″ that is intercepted by surface ″*j*″. This is determined by the view factor $F_{i_{j}}$ , calculated using MCRT for each interior tube configuration.
11 $$Q_{{\mathrm{rad}}_{j}}=\overset{n}{\underset{i}{\sum }}F_{i_{j}}S_{i}\sigma \epsilon_{i}T_{i}^{4}$$
  Here, *S*_*i*_ is the emitting surface area of element *i*, ϵ_*i*_ is emissivity, *T*_*i*_ is temperature, and σ is the Stefan–Boltzmann constant (5.670367 × 10^–8^ *W*/*m* ^2^ *K* ^4^).
3 Emitted Radiation $\left(Q_{em_{j}}\right)$ is the thermal radiation emitted by surface ″*j*″ based on its own temperature *T*_*j*_.
12 $$Q_{em_{j}}=S_{j}\sigma \epsilon_{j}T_{j}^{4}$$
4 Losses $\left(q_{net_{j}}\right)$ , this term is composed of three distinct parts:
  Convection to the air inside the cavity $\left(Q_{conv,cav_{j}}\right)$ :
13 $$Q_{conv,cav_{j}}=h_{k}S_{j}\left(T_{j}-T_{amb,cav}\right)$$
    Where *h*_*k*_ and *T*_amb,cav_ are the convection heat transfer coefficient and the air temperature inside the receiver.
  Convection to the ambient from the receiver’s exterior walls $\left(Q_{conv,amb_{j}}\right)$ , which only applies to the outer cylinder walls (*j = 1* and *j = 2*):
14 $$Q_{conv,amb_{j}}=US_{j}\left(T_{j}-T_{amb}\right)$$
    Here, *U* is the external heat transfer coefficient (0.128 *W*/*m* ^2^ *K*)^23^ and *T*_amb_ is the ambient temperature outside the receiver.
  The complex internal heat transfer, including fluid convection, conduction through the tube walls, and the chemical reaction kinetics is simplified into a single heat utilization factor (1 - ξ), representing the fraction of incident energy successfully used by the process. This decoupling of the external thermal-optical model from the internal chemical processes is a standard methodology used to assess overall system feasibility. The model therefore solves for the steady-state outer tube wall temperature (*T*_*j*_) under a representative heat load.^19,48^ According to the Second Law of Thermodynamics, for the working fluid to reach the target reaction temperature >1300 °C, the tube wall temperature must be higher. Thus, demonstrating that *T*_*j*_ can achieve or exceed this temperature confirms that the process is thermodynamically viable.

#### Numerical Solution

The system consists of 6,242 coupled, nonlinear energy balance equations that are solved using an iterative numerical approach. The simulation begins with an initial temperature vector (*T*_*j*_) assigned to all surface elements.

In each iteration, the model solves the primary energy balance for each surface, denoted as *j*. This balance requires that the net energy available at the surface equals the energy lost through emission and convection:

15 $$\left(1-\xi \right)\left(\mu_{Nh}P_{h_{j}}+\overset{n}{\underset{i}{\sum }}F_{i,j}S_{i}\sigma \epsilon_{i}T_{i}^{4}\right)=S_{j}\sigma \epsilon_{j}T_{j}^{4}+h_{k}S_{j}\left(T_{j}-T_{ambr}\right)+US_{j}\left(T_{j}-T_{amb}\right)$$

To manage the nonlinearity of the equations, the incident radiation term on the left, which depends on neighboring temperatures (*T*_*i*_), and the convective terms on the right are calculated using the temperature values from the previous iteration. This approach allows the equation to be rearranged algebraically and solved directly for updated temperature vectors, *T*_*jnew*_ and *T*_*amb,cav*_. This process continues until the maximum relative difference between the temperature vectors of successive iterations falls below a specified convergence tolerance. At that point, the final steady-state temperatures are obtained.

## Results and Discussion

### Absorption of Solar Energy

To determine the power contribution factor (*Ph*_*j*_, eq 10) from each heliostat, we performed a 3D Monte Carlo ray-tracing simulation of 1.1 × 10^8^ photon bundles. The model assumed a heliostat optical error of 2 *mrad*, resulting in *Ph*_*j*_ values with standard deviations below 4%. Figure 4 illustrates the resulting incident solar power distribution in the cavity aperture. We validated our MATLAB model against SolTrace, which confirmed the accuracy of our results.

**Figure 4:**
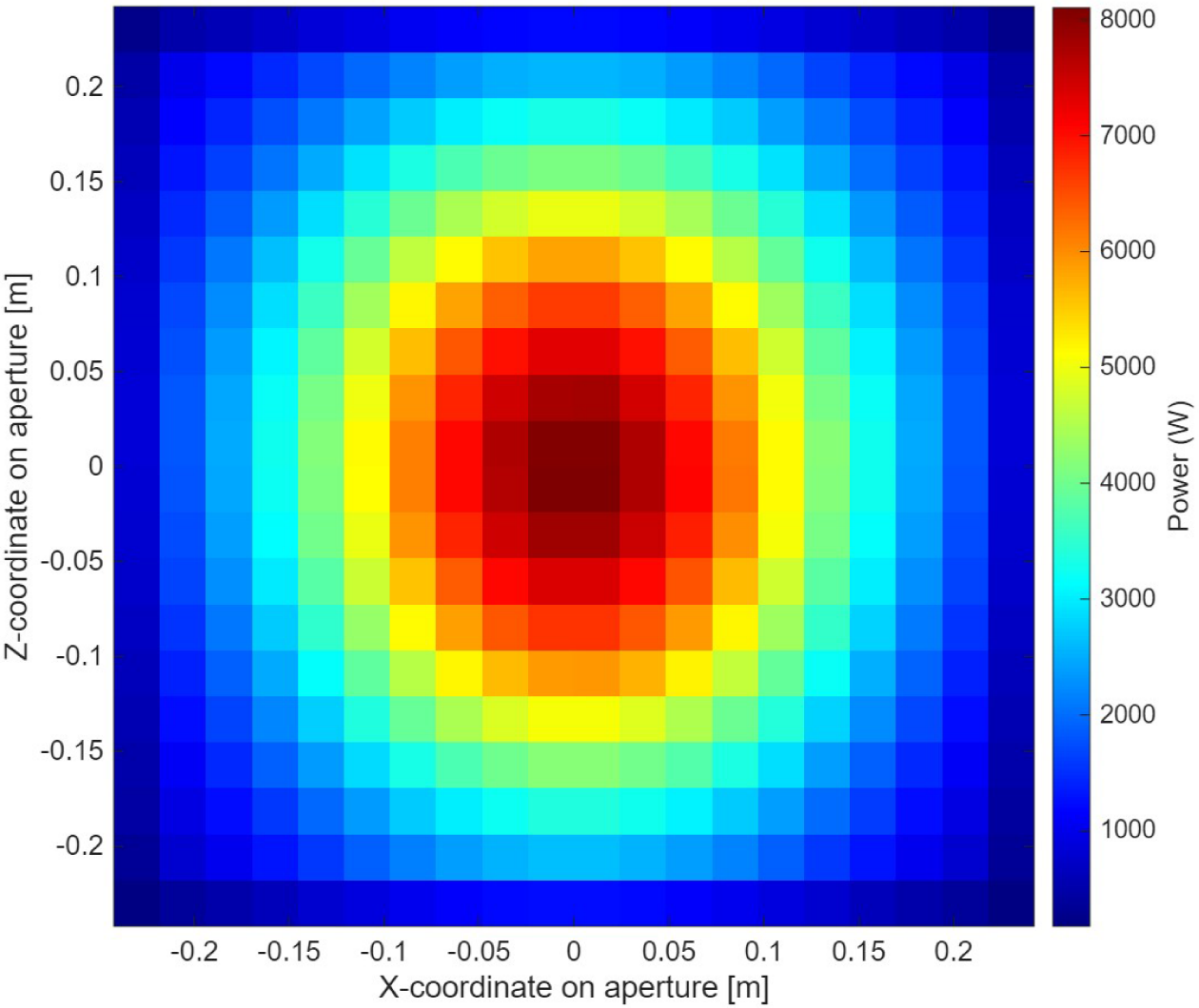
Incident solar power [*W*] distribution in the cavity aperture (heliostat optical error of 2 mrad).

The solar intercept factor was approximately 79%, and the solar thermal power accessing the cavity was 1.25 MWth, with a standard deviation of 0.01%.

Photons traveling from the heliostats that enter the cavity through the receivers’ aperture can be absorbed or diffusely reflected (max 5 times) from the ″*j*″ inner elements. If reflected, the photon can be absorbed by another ″*j*″ element or exit the cavity through the aperture.

The distributions of average solar flux (*Q*_*solarabs*_) absorbed by all tubes for four different arrangements inside the cavity receiver are shown in Figure 5. These profiles are analyzed to identify a configuration that maximizes thermal power while ensuring long-term reliability of the receiver, specifically considering how the geometric layout interacts with the concentrated solar flux entering the cavity.

**Figure 5:**
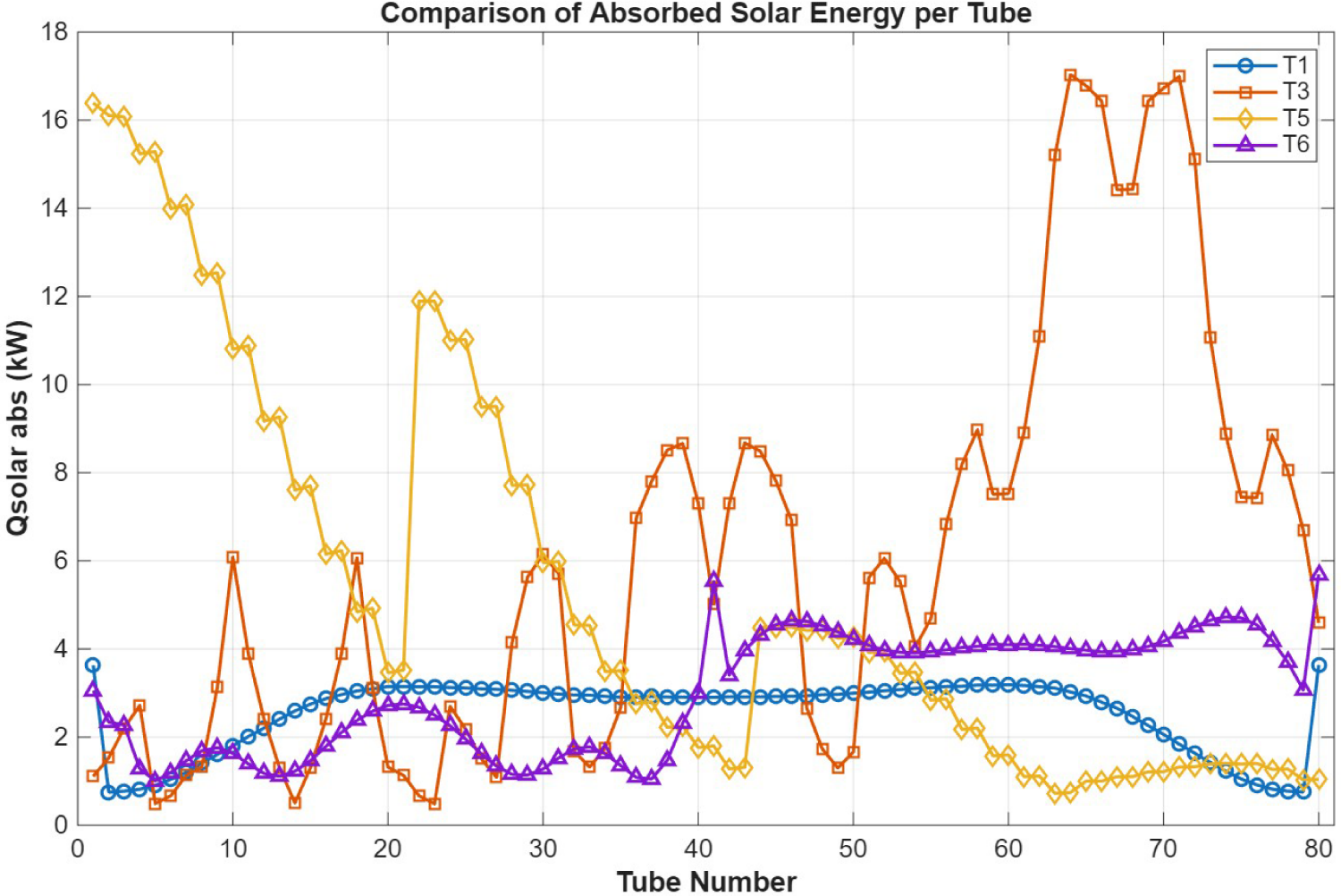
Total solar energy flux absorbed by TC1, TC3, TC5, and TC6.

The TC3 configuration (orange squares) employs a multilayered zigzag arrangement, resulting in an aggressive absorption profile with sharp, high-intensity peaks of approximately 17 kW. This arrangement produces pronounced power gradients and shading effects, especially between tubes 60 and 80. Similarly, TC5 (yellow diamonds) adopts a three-row horizontal layout that concentrates energy at the cavity entrance, with initial power levels near 16.5 kW. Absorption declines substantially after a secondary peak of 12 kW at tube 20. These resulting power gradients lead to severe thermal differences across the tube walls, causing high thermal stress that accelerates material degradation through creep and fatigue, ultimately reducing the receiver’s operational lifetime.^49^

Circular geometries, such as the single-layer TC1 (blue circles), produce more stable absorption profiles by conforming to the cavity wall, limiting peak power to 3.2 kW. This power is distributed broadly and evenly across a wide section of the receiver. This tube configuration successfully minimizes thermal gradients, which in turn reduces associated mechanical stresses. As a result, this approach could guarantee the long-term structural integrity and reliability of the receiver, a crucial factor for the overall economic viability of the plant. TC6 (purple triangles) utilizes a double-layer staggered circular geometry that balances performance and reliability. It achieves moderate peaks of 5.8 kW and maintains stable intermediate absorption at 4 kW, delivering a higher total power than TC1 while avoiding the severe stress concentrations present in TC3.

### Analysis of Thermal Power Distribution Inside the Solar Cavity

To analyze radiative heat transfer with high fidelity, we used the Monte Carlo ray tracing method, tracing 8 billion photon bundles within the cavity receiver. We validated our MATLAB model against COMSOL Multiphysics. Using the ″Finer″ mesh setting, the results showed close agreement, with a mean standard deviation below 1%. A simplification for convective losses was applied, ensuring both precision and computational efficiency.^50^ We assume that the temperature inside the cavity matches the mean temperature of the inner surfaces. Convection losses are calculated using eq 13, with a convection coefficient of *h* = 6 W/m^2^K corresponding to forced convection at fluid velocities of approximately 1–3 m/s.^51,52^ The subsequent results correspond to a heat extraction parameter value of (1 - ξ) = 0.5. Where, (1 - ξ) ranges from 1 (ξ = 0, no heat removal) to 0 (ξ = 1, maximum heat removal).

Analysis of the energy distribution for configurations TC1 through TC6 indicates that TC1 is the most thermally efficient design in this group, achieving the highest useful energy conversion fraction (*Q*_*inner‑tube*_) at 24%, as shown in Figure 6. Consequently, TC1 exhibits the lowest relative thermal losses. In contrast, TC2 and TC4 are the least efficient configurations, with useful energy fractions of 20% and 21%, respectively, and the highest combined thermal losses.

**Figure 6:**
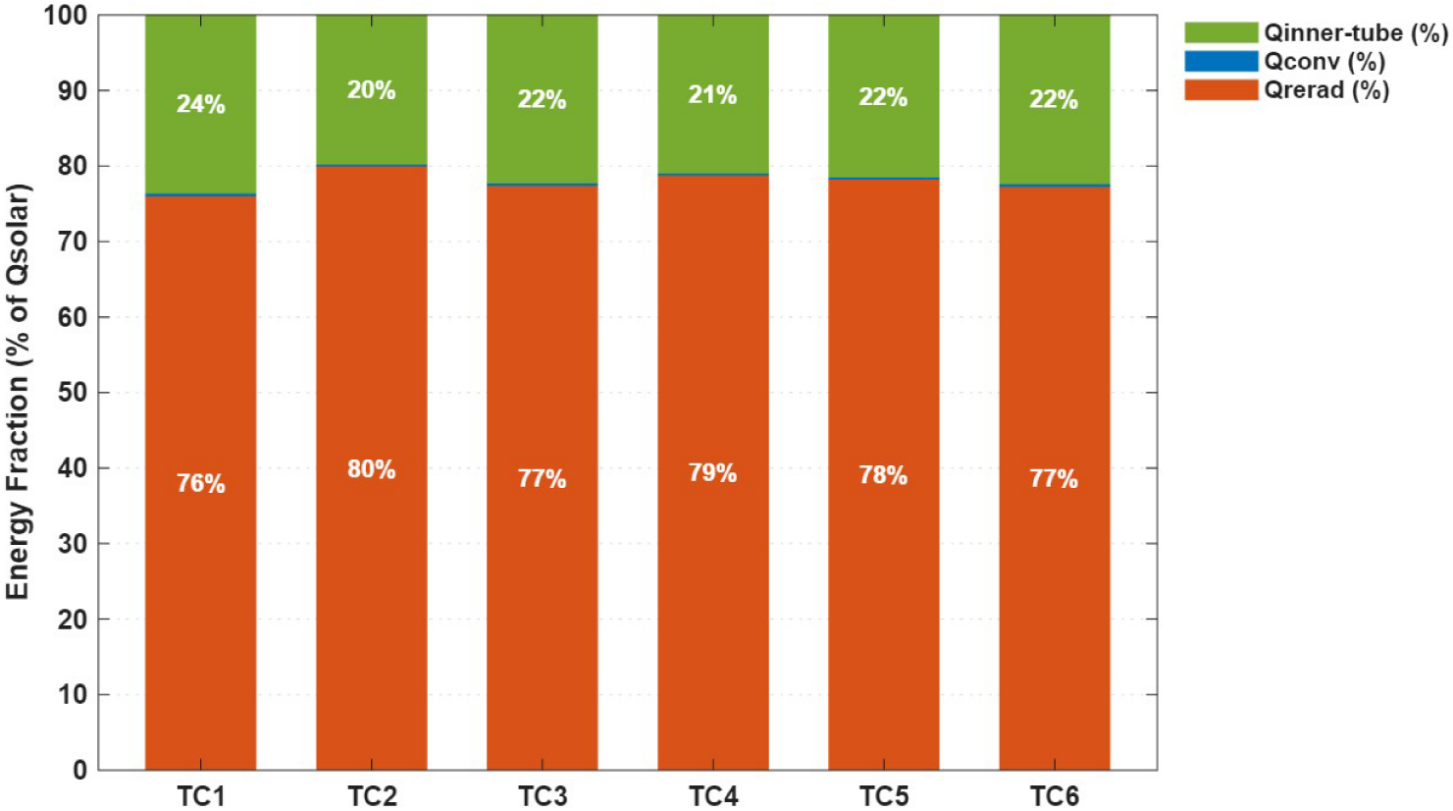
Energy balance for each tube configuration as a fraction of absorbed solar power.

For all evaluated configurations (TC1–TC6), reradiation (*Q*_*rerad*_) remains the dominant mode of heat loss, representing 76% to 80% of total solar energy (*Q*_*solar*_). Convective losses (*Q*_*conv*_) are negligible and contribute only minimally to the energy balance. These findings confirm that radiative heat transfer governs the thermal behavior of the system,^19^ and performance differences arise from the capacity of each geometry to reduce radiative losses.

### Temperature Distributions

Based on the data in Figure 7, configurations TC1 and TC6 emerge as the optimal operational choices, achieving the highest mean temperatures of 1426.6 °C and 1427.7 °C, respectively, while maintaining the lowest Coefficient of Variation (CV), ∼ 0.12.

**Figure 7:**
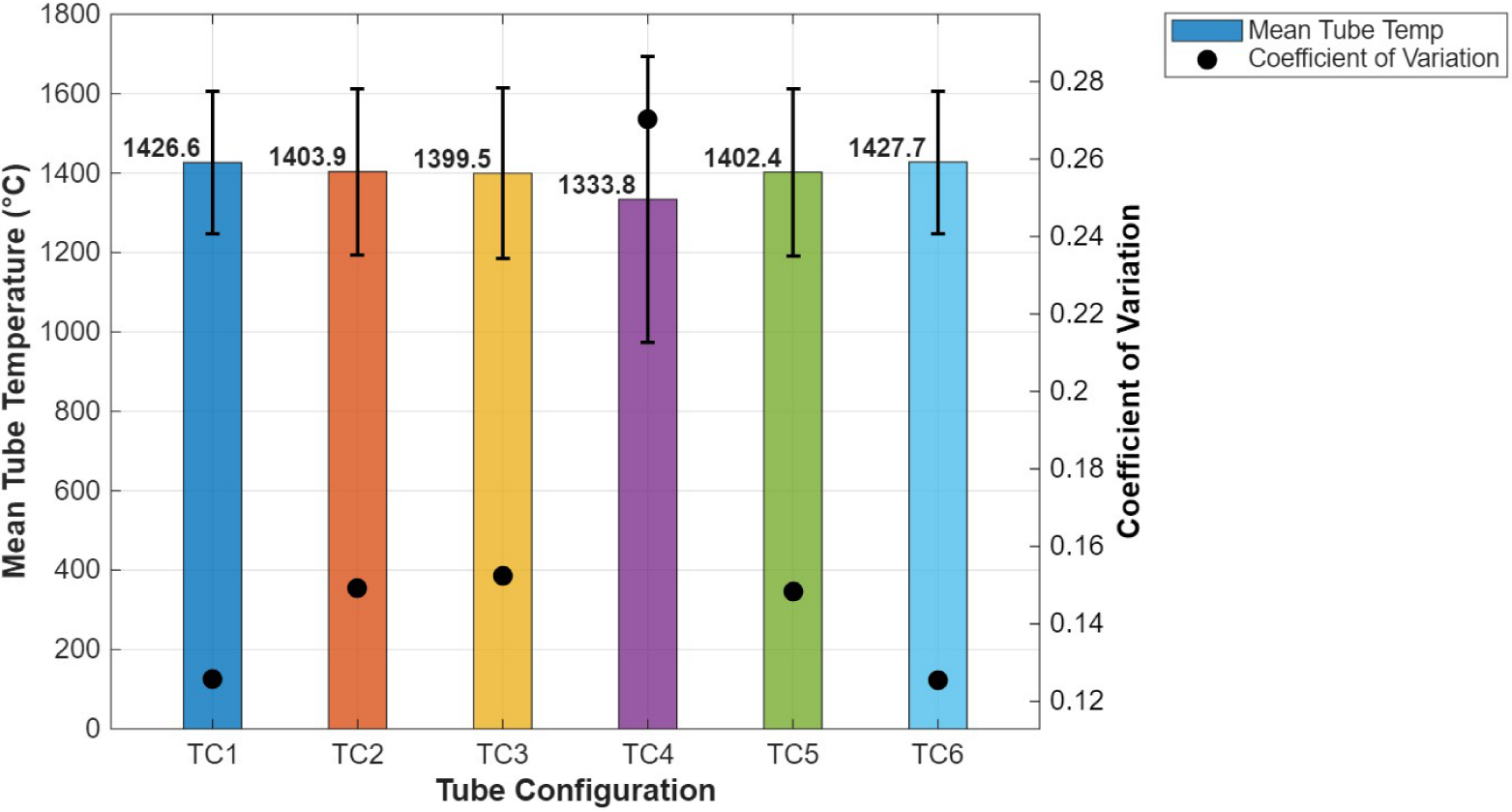
Mean tube temperature and Coefficient of Variation for six tube configurations.

This low variability is critical because it prevents thermal shocks in the alumina tubes.^19^ TC4 is the least viable option in this set, recording the lowest mean temperature (1333.8 °C) and the highest nonuniformity (CV > 0.25); such large deviations suggest unstable heat flux distribution, which hinders the consistent performance required for effective receiver operation.

Although all configurations exceed the required minimum temperature of 1300 °C, TC1 and TC6 balance high temperature with thermal stability. Operating at these elevated temperatures benefits reaction kinetics and chemical conversion rates, but must be managed carefully to reduce heat losses, particularly reradiation, which becomes predominant at higher temperatures, as described by the Stefan–Boltzmann law.^19^ The superior uniformity of TC1 and TC6 minimizes the risks of localized overheating or underheating observed in TC4 and the intermediate configurations (TC2, TC3, TC5), ensuring a more efficient and reliable continuous thermochemical process.

TC2 demonstrates the highest thermal potential among the system configurations, achieving peak tube temperatures of approximately 1650 °C, as shown in Figure 8. The staggered, multirow arrangement maximizes the capture of incident radiation in specific zones, likely due to reradiation effects within the deeper tube field. However, this configuration exhibits poor uniformity, as the temperature distribution is discontinuous and patchy, with sharp gradients between adjacent tubes in different rows. These findings indicate that, despite high energy absorption, the sparse and scattered nature of the array results in significant shading and uneven flux distribution.

**Figure 8:**
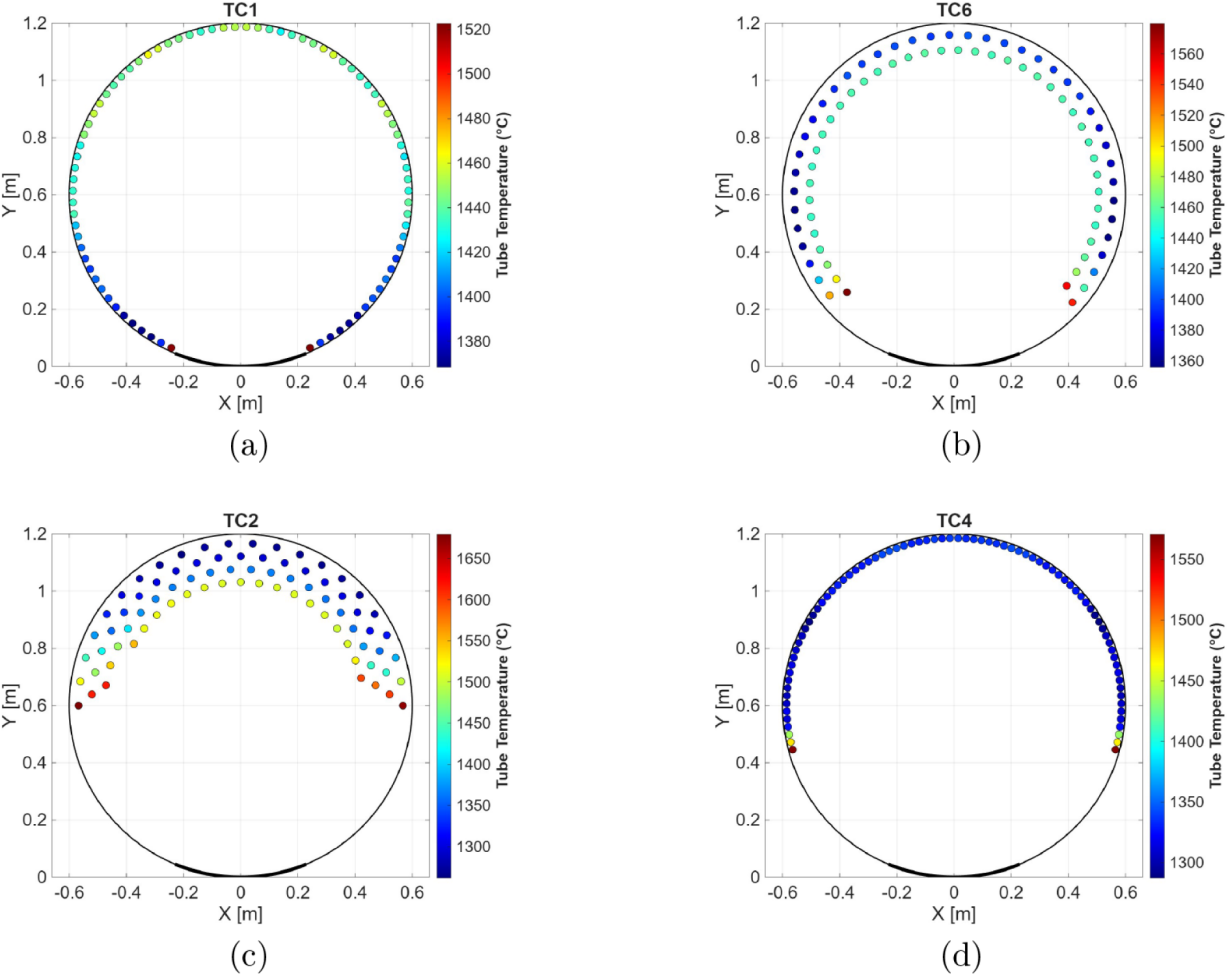
Temperature distribution inside the reactor for (a) TC1, (b) TC6, (c) TC2 and (d) TC4.

In contrast, TC1 presents the most homogeneous temperature profile, providing a stable thermal distribution despite a high peak temperature of approximately 1520 °C. The geometry, which consists of a single, tightly packed continuous arc, facilitates a smooth temperature transition from the hotter tips near the aperture to the cooler upper section. This arrangement avoids the steep radial gradients observed in multirow designs. Although TC1 may not capture as much total energy as TC2, it distributes absorbed heat more effectively across the available surface area, thereby minimizing thermal stress risks.

TC6 and TC4 display distinct limitations in interception efficiency. TC6, which utilizes a concentric double-row layout, achieves moderate peak temperatures of 1560 °C but introduces a clear radial disparity, as the inner row maintains consistently higher temperatures than the outer row. TC4 exhibits the lowest overall performance; although the tips reach 1550 °C, the majority of the upper arc remains at a minimum temperature of approximately 1300 °C. This outcome indicates that the TC4 geometry causes a large portion of the receiver area to contribute negligibly to energy absorption.

Thermochemical cycles for hydrogen production operate within specific high-temperature ranges, depending on the metal oxide employed. For example, the ceria (*CeO*_2_) cycle involves a nonstoichiometric reduction step at temperatures ranging from 1400 to 1600 °C, followed by an oxidation step with water at 900 to 1200 °C.^53^ Similarly, the iron oxide (*Fe*_3_ *O*_4_) cycle requires reduction temperatures of up to 1600 °C (or even 1200 °C for hercynite variants) and a subsequent oxidation step around 1000 °C.^54^ More demanding cycles, such as the zinc oxide (ZnO) process, require a much higher reduction temperature of approximately 2027 °C, while the corresponding hydrolysis step occurs at 427 °C.^48^

Figure 9 shows the relationship between the mean tube temperature and the heat removal coefficient (ξ) for six system configurations (TC1–TC6). The *y*-axis, (1-ξ), reflects heat extraction, where a value of 1 means that there is no heat removal (ξ = 0) and the highest tube temperature, while 0 indicates maximum heat removal (ξ = 1).

**Figure 9:**
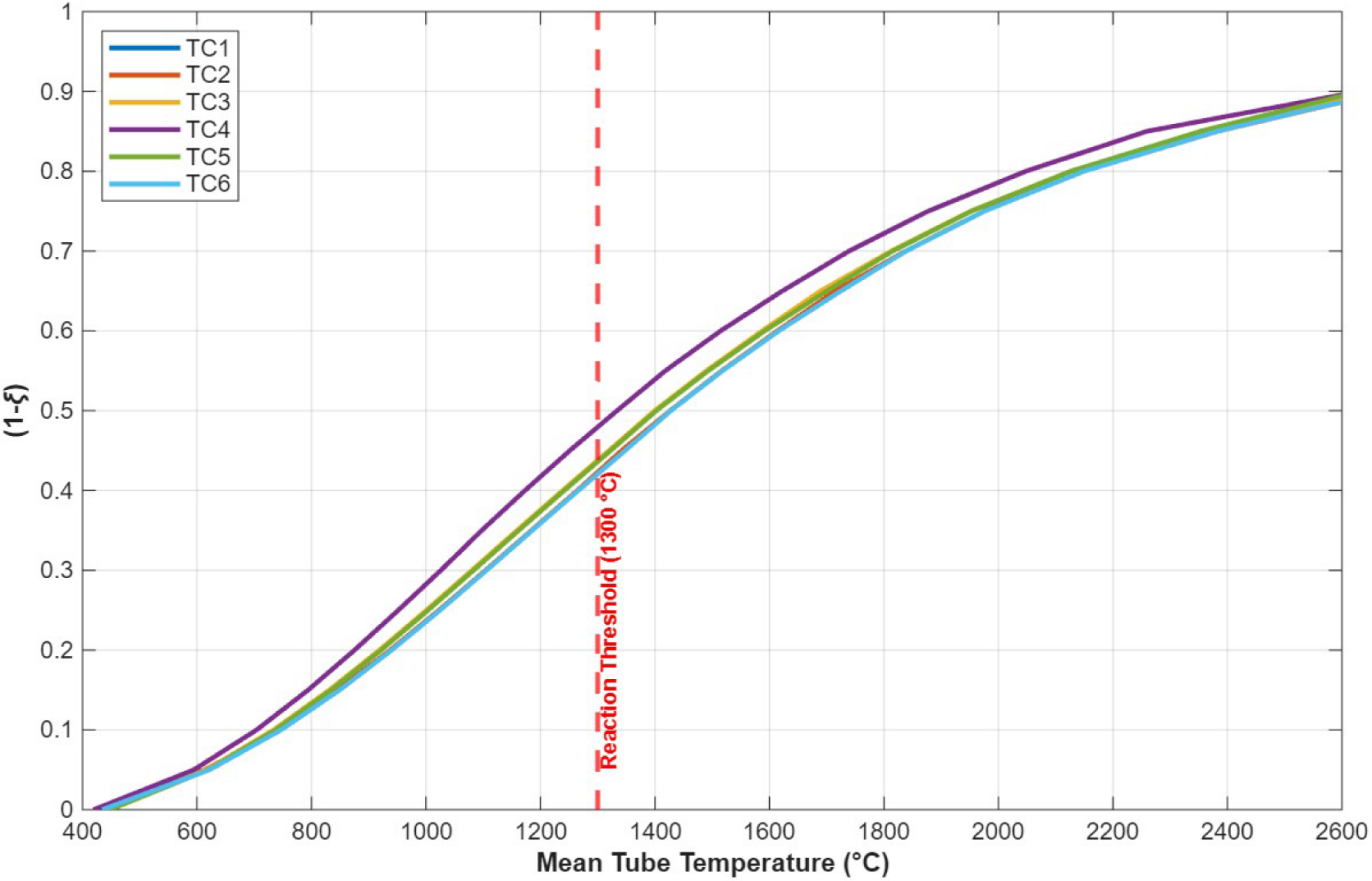
Maximum temperature [°C] for (1-ξ)= 0:0.01:0.9 value.

A critical threshold at 1300 °C is required for the chemical reaction to occur. The viability of each configuration is limited to the area right of this threshold, with the intersection of each curve and the 1300 °C line marking the maximum permissible heat removal coefficient (ξ_*max*_) for that configuration. For the reaction to proceed, the actual heat removal (ξ) must be less than or equal to ξ_*max*_; otherwise, the tube temperature will fall below 1300 °C, halting the reaction and reducing the efficiency of the process.

According to the second law of thermodynamics, heat can only be transferred from a higher-temperature body to a lower-temperature one. Therefore, for these endothermic reduction reactions to take place, the heat source must be at a temperature equal to or greater than the required reaction temperature. The previously analyzed tube configurations, which achieved temperatures exceeding 2000 °C, demonstrate the capacity to provide the thermal energy necessary to drive demanding cycles like the ZnO process.

The analysis indicates that TC1 (dark blue) and TC6 (cyan) are the most efficient configurations. Because their performance is nearly identical, the cyan line largely overlaps the blue line in Figure 7. At the threshold 1300 °C, both configurations achieve the lowest value (1-ξ) of approximately 0.42. This results in the highest allowable heat removal capacity (ξ ≈ 0.58), enabling the system to maintain the required reaction temperature while effectively transferring heat to the working fluid.

In contrast, TC4 (purple line) represents the upper bound of the plotted data and is the least effective configuration in this group. At the threshold, it has a higher (1-ξ) value of approximately 0.48, corresponding to a lower heat removal coefficient (ξ ≈ 0.52). This restricts the system’s capacity to extract heat without dropping below the critical temperature. Therefore, selecting the TC1/TC6 configuration offers a clear thermodynamic advantage in achieving optimal performance in high-temperature cycles.

### Comparative Analysis with Existing Simulated Receivers

To fully evaluate the performance of the proposed optimized cavity geometry, the simulated thermal distribution of this multitubular cavity receiver was evaluated against existing numerical models of solar receivers, as detailed in Table 2. A persistent challenge identified in simulated models within the literature is the occurrence of severe thermal gradients. Recent multitubular models by Sánchez-Mora et al.,^24^ Tapia et al.^23^ and Li et al.^56^ accurately represent complex heat transfer phenomena, yet continue to report localized hot spots and significant Δ*T* values across tube arrays. Similarly, earlier numerical analyses of cavity receivers (e.g., Martinek et al.,^32^ Valades-Pelayo et al.^34^) indicate that, in the absence of rigorous geometric optimization, simulated components experience nonuniform flux profiles that can induce critical thermal stresses in practical applications.

**Table 2:** Comparison of Various Receiver Systems, Modeling Approaches, and Temperature Uniformities

System & Reference	Receiver Type & Scale	Max Temp (exp./sim.)	Simulated Temperature Uniformity	Modeling Approach
HFSS (ETH)^19^	Cylindrical Cavity with Al_2_O_3_ tubes perpendicular to optical axis (40 kW/m)	1975 K/2300 K	Validated: Mean ΔT between measured and calculated 4.61% ±4.87%. Inner-outer absorber ΔT < 12 K	2D steady-state Finite Difference + MCRT
SSPS-CRS (PSA)^23^	Semicylindrical; 80 staggered tubes perpendicular to optical axis (80 kWth)	1473 K/>1473 K	Moderate gradients: Δ*T* = 120–140 K between average temperature of tubes. Error 1–10%. Max. ΔT between tubes 300 K	3D computational fluid dynamics CFD (ANSYS) + MCRT
HFSF (NREL)^32^	Windowed cylindrical; 5 flow tubes perpendicular to optical axis (6 kWth)	—/1820 K	Nonuniform: Vertical Δ*T* = 450 K over 5 cm; horizontal Δ*T* = 340 K between front and back of center tube.	3D CFD + Finite Volume + MCRT
HoSIER (UNAM)^34^	Cubic cavity; multitubular, perpendicular to optical axis (25 kWth)	—/2798 K	Highly uniform (Optimized): Max single tube temperature standard deviation ≤ 80 K. Unoptimized geometries with severe hotspots (Max. SD > 300 K)	Steady-state + Hybrid Monte Carlo-Finite Volume Method
HoSIER (UNAM)^24^	Cubic cavity; multitubular, perpendicular to optical axis (25 kWth)	1430 K/∼1430 K	Nonuniform: Gaussian distribution; temperature highly concentrated in the middle of the tubes. Max. error 7.4% between experimental and calculated. ΔT ∼ 500 K over 10 cm	2D transient Finite Difference + Matrix method
HFSS (UF)^56^	Cylindrical cavity with 14 tubular absorbers parallel to optical axis (10 kWth)	—/1843 K	Longitudinal nonuniformity: Central hot spot; ΔT ∼ 200 K. Azimuthally uniform as heating progresses.	Lattice Boltzmann (LB) + MCRT
HFSS (UMN)^57^	Cylindrical cavity; concentric alumina tubes parallel to optical axis (3 kWth)	—/1813 K	Highly uniform: Radial Δ*T* = 2–4 K; maximum circumferential ΔT ≈ 8 K.	1D Finite Volume (FORTRAN) + ANSYS + MCRT
Present Work	Cylindrical cavity; 80 alumina tubes perpendicular to optical axis (1.5 MWth)	—/∼ 1923 K	Highly controlled and stable (Optimized): CV ≈ 0.12% avoiding steep radial gradients. Unoptimized geometries show sharp gradients.	3D steady-state numerical iterative + MCRT (MATLAB)

Accurate modeling of heat transfer and energy transport is fundamental to solar reactor design, offering critical insights for optimizing operational parameters.^56^ The internal arrangement of tubes within these reactors plays a pivotal role, as both radial temperature uniformity and overall thermal stability are strongly influenced by this configuration.^24^ When earlier simulations revealed excessive temperature gradients and highly nonuniform distributions, researchers investigated a range of design modifications to address these challenges. For example, Martinek et al.^32^ demonstrated that strategic removal or repositioning of tubes can enhance specular reflections and reduce temperature discrepancies between the front and back of the reactor. Additional optimization strategies have included adjusting the cavity aperture size (Melchior et al.),^19^ modifying the cavity radius in conjunction with tube row arrangements to eliminate temperature differences (Tapia et al.)^23^ and focusing on the geometric optimization of tube locations to improve thermal performance (Valades-Pelayo et al.).^34^ Although previous studies have investigated variations in opening sizes, cavity volumes, materials, tube radii, and tube arrangements (such optimizations have typically been limited to arrays with fewer than 20 tubes). The present study demonstrates that, for a cavity receiver with constant size, form, material, and opening, changes in tube array configuration alone can significantly affect maximum temperatures and the overall quality of temperature distribution. Optimization of the geometric parameters in a large-scale array of 80 vertical tubes mitigates the pronounced Gaussian flux distributions frequently reported in the literature. The resulting effectiveness indices, particularly the reduction of steep radial and longitudinal gradients to a coefficient of variation of approximately 0.12, indicate a highly controlled thermal environment. This stable uniformity constitutes a significant advancement.

## Conclusions

A validated three-dimensional computational heat transfer model, incorporating Monte Carlo ray-tracing, was used to evaluate six different tube configurations within a solar cavity receiver for a 1.5 MWth solar tower plant. The results indicate that the geometric arrangement of the tubes is a key design parameter that directly influences temperature distribution and thermal efficiency by modulating heat-loss mechanisms.

Based on a comprehensive analysis, the geometric configuration of a solar receiver is one of the most important design parameters that determines its thermodynamic efficiency and sets the operational limits for a specific thermochemical process.

In this work, reradiation through the aperture and within the cavity was identified as the dominant heat loss mechanism across all configurations, accounting for 76% to 80% of the total incident solar energy, consistent with previous cavity-receiver literature. These findings validate the necessity of geometric optimization to maximize capture efficiency, since reradiation losses increase substantially at the high temperatures required for thermochemical cycles.

The single-layer circular arrangement (TC1) and the double-layer staggered configuration (TC6) were identified as the most efficient designs. TC1 achieved the highest useful energy conversion fraction (24%) and exhibited the lowest relative thermal losses. Both configurations maintained high mean temperatures (approximately 1427 °C) with an exceptionally low coefficient of variation (CV ≈ 0.12%). Such uniformity is critical, as statistical indices of heat flux distribution are directly correlated with collector thermal efficiency and outlet temperatures.

Although the staggered multirow configuration (TC2) achieved the highest peak temperatures (approximately 1650 °C), it exhibited significant nonuniformity, resulting in a discontinuous temperature distribution. High peak temperatures are insufficient if accompanied by severe thermal gradients, as they can cause material degradation. Studies of multitubular reactors have demonstrated that maintaining a smooth temperature profile is essential to reduce thermal stresses and ensure mechanical durability.

Analysis of the heat removal coefficient (ξ) indicated that TC1 and TC6 provide the highest operational flexibility. Both configurations support a heat removal coefficient of ξ ≈ 0.58 while maintaining tube wall temperatures above the critical threshold of 1300 °C required for the reduction step. In contrast, TC4 exhibited the lowest performance.

The results demonstrate that a uniform, closely spaced tube layout (TC1) is superior to complex multilayered arrangements (TC3, TC5) for this specific cavity scale. Minimizing shading and optimizing view factors in the TC1 design reduces the risk of localized overheating and maximizes solar-to-chemical energy conversion potential. This work confirms that specific geometric arrangements enable the scaling up of thermochemical reactors in solar tower plants.

Future research should couple the optical-thermal model with reaction kinetics to simulate transient states and investigate the integration of spectrally selective coatings to further mitigate the dominant reradiation losses identified in this study.

In summary, this study demonstrates that effective reactor design should prioritize a balanced approach to achieve specific process temperatures while minimizing heat losses, rather than solely maximizing peak temperatures.
